# Semi-Automated Determination of Heavy Metals in Autopsy Tissue Using Robot-Assisted Sample Preparation and ICP-MS

**DOI:** 10.3390/molecules26133820

**Published:** 2021-06-23

**Authors:** Heidi Fleischer, Christoph Lutter, Andreas Büttner, Wolfram Mittelmeier, Kerstin Thurow

**Affiliations:** 1 Institute of Automation, University of Rostock, 18119 Rostock, Germany; 2 Department of Orthopedics, University Medical Center, 18057 Rostock, Germany; christoph.lutter@uni-rostock.de (C.L.); wolfram.mittelmeier@uni-rostock.de (W.M.); 3 Institute of Legal Medicine, University Medical Center, 18055 Rostock, Germany; andreas.buettner@med.uni-rostock.de; 4 Center for Life Science Automation (Celisca), University of Rostock, 18119 Rostock, Germany; kerstin.thurow@uni-rostock.de

**Keywords:** automated measurements, compound-oriented measurements, robot-assisted sample preparation, heavy metals, hip implant, knee implant, periprosthetic autopsy tissue, ICP-MS

## Abstract

The endoprosthetic care of hip and knee joints introduces multiple materials into the human body. Metal containing implant surfaces release degradation products such as particulate wear and corrosion debris, metal-protein complexes, free metallic ions, inorganic metal salts or oxides. Depending on the material composition of the prostheses, a systemic exposure occurs and may result in increasing metal concentrations in body fluids and tissues especially in the case of malfunctions of the arthroplasty components. High concentrations of Cr, Co, Ni, Ti and Al affect multiple organs such as thyroid, heart, lung and cranial nerves and may lead to metallosis, intoxications, poly-neuropathy, retinopathy, cardiomyopathy and the formation of localized pseudo tumors. The determination of the concentration of metals in body fluids and tissues can be used for predicting failure of hip or knee replacements to prevent subsequent severe intoxications. A semi-automated robot-assisted measurement system is presented for the determination of heavy metals in human tissue samples using inductively coupled plasma mass spectrometry (ICP-MS). The manual and automated measurement processes were similarly validated using certified reference material and the results are compared and discussed. The automation system was successfully applied in the determination of heavy metals in human tissue; the first results are presented.

## 1. Introduction

Hip and knee joint arthroplasty represent common treatment options for severe arthritis in both elderly and younger patients [1]. Different types of bearing materials and combinations are used such as metal-on-polyethylene (MOP), ceramic-on-polyethylene (COP), ceramic-on-ceramic (COC) and metal-on-metal (MOM) [2,3]. Since prostheses usually have metallic components, they release degradation products such as particulate wear and corrosion debris, metal-protein complexes, free metallic ions, inorganic metal salts or oxides, and/or metals sequestered in an organic storage form [2]. Depending on the material composition of the prostheses, a systemic exposure to chromium (Cr), cobalt (Co), nickel (Ni), molybdenum (Mo), vanadium (V), titanium (Ti) and aluminum (Al) alloys occurs and may result in increasing metal concentrations in biological fluids and various organs especially in the case of malfunctions of the joint implants [2,3,4].

High concentrations of Cr, Co, Ni, and Al may result in numerous toxic effects. Implant malfunctions can lead to an increased Ni, Co and/or Cr sensitivity (hypersensitivity), skin rashes such as local or generalized skin eczema, wound healing disorders, recurrent pain, hematoma, limited mobility or even implant loosening [5,6]. Carcinogenicity and fetal exposure to metallic ions in pregnancy are further documented effects of prolonged exposure to increased concentrations of metal ions [7].

The determination of the concentration of metals in body fluids and tissues can be used for predicting failure of hip or knee replacements to prevent subsequent severe intoxications. Hart et al. presented in their study the sensitivity and specificity of Co and Cr values in blood to distinguish between well-working and failed hip replacements [8]. Several studies reported the determination of the metal concentration in body fluids and tissues using different sample sources, sampling methods and measurement techniques (see Section 2). The number of samples is still limited and the measurement results are difficult to compare due to the variations of the experiments. Furthermore, the sample preparation procedures are often time consuming and characterized by a high human workload forming a bottleneck in the realization of high-throughput measurements.

The failure of metal-containing orthopedic devices is one way of introducting multiple metals into the body. There are further pathways that can also result in elevated metal levels in tissues such as the ingestion of metals via drinking water and nutrition, envi-ronmental conditions, accidental and occupational exposures, pharmaceuticals as well as personal life style (e.g., smoking) [9,10]. A widespread examination of a high number of human samples is required to study the effects of metal debris caused by various kinds of hip and knee implants. Therefore, suitable sample preparation procedures and measurement techniques are required which allow a high sample throughput and provide a similar or even better precision and trueness compared to manual processing.

In this study, an semi-automated robot-assisted measurement system is presented for the determination of heavy metals in human tissue samples using inductively coupled plasma mass spectrometry (ICP-MS). In Section 2, common analytical measurement techniques for the determination of heavy metals in human and animal tissues and body fluids are presented. The advantages of ICP-MS measurements are highlighted for the aim of this study. Section 3 explains the general measurement process and introduces the system concept and design of the developed robot-assisted automation system. The validation results of the manual and the automated measurement processes are presented in Section 4 and discussed in Section 5. Measurement results of the first human samples are given in Section 6. In the last section, the materials and measurement method details are provided. The paper concludes with a summary and an outlook to further investigations.

## 2. Determination of Heavy Metals in Human and Animal Tissues

Numerous analytical measurement techniques are available for the determination of metals in biological samples. The most frequently used techniques are flame and graphite furnace atomic absorption spectroscopy (F-AAS, GF-AAS), inductively coupled plasma optical emission spectroscopy (ICP-OES) and, especially, inductively coupled plasma mass spectrometry (ICP-MS) [11,12,13,14,15]. Furthermore, measurements using optical microscopy, transmission electron microscopy (TEM), radiochemical neutron activation analysis (RNAA), instrumental neutron activation analysis (INAA), micro X-ray fluorescence (µ-XRF) elemental mapping, and microfocus X-ray absorption spectroscopy (µ-XAS) are documented [16,17,18].

The selection of a suitable analytical measurement technique always depends on the elements to be determined, the sample matrix (e.g., blood, urine, tissue, bone)—which may require a complex sample preparation if necessary—and the aim of investigation. The following examples will give an overview about the recently used measurement procedures in this area.

The determination of essential and toxic trace metals in human autopsy tissues to examine the role of toxic elements such as lead (Pb), cadmium (Cd) and mercury (Hg) was presented by Bush et al. using three different analytical techniques [19]. Essential elements including calcium (Ca), copper (Cu), iron (Fe), magnesium (Mg), and zinc (Zn) were measured by ICP-OES, heavy metals including cadmium (Cd), manganese (Mn), arsenic (As) and lead (Pb) by GF-AAS, mercury (Hg) by cold vapor AAS and aluminum (Al) by ICP-MS. Prior the measurement, the tissue samples were digested in Teflon vessels at a heating block at 95 °C (203 °F) over 12 h using nitric acid and hydrogen peroxide. The certified reference materials NIST 1577a and NRC Canada DORM-1 were used to determine precision and accuracy. A specialty of this study is the comparison of the measurement of fresh and formalin-fixed tissue samples. No significant influence was reported for the majority of elements measured. Influences were found for calcium (Ca) and magnesium (Mg) from samples in phosphate-buffered formalin solutions. Furthermore, an increasing concentration of aluminum (Al) and a decreasing concentration of manganese (Mn) were observed with an increasing storage time [19].

The determination of heavy metals in formalin-fixed brain tissue to study neurogenerative disorders was documented by Gellein et al. [20]. Brain tissue samples with a wet weight of 0.5–1.0 g were freeze-dried until constant weight and digested with 2 mL nitric acid in Teflon vessels using a microwave oven. After appropriate dilution, the analysis was performed using double-focusing magnetic sector field ICP-MS. The results were supported using the certified reference material NIST 1577b (bovine liver). Furthermore, the heavy metal concentrations were determined in fresh unused formalin and in sample storage solutions to study the effects of sample storage. An increasing concentration of the elements measured was documented with increasing storage period over several years. It was concluded that this leaching effect results in too low element concentrations [20]. Meldrum et al. also describe observed erroneous aluminum (Al) and cobalt (Co) concentrations if the tissue was previously treated using formalin [21].

The concentrations of cobalt (Co), chromium (Cr), antimony (Sb) and scandium (Sr) were determined in tissues of various organs (heart, kidney, liver and spleen) by Schnabel et al. using radiochemical neutron activation analysis (RNAA) and instrumental neutron activation analysis (INAA) [17]. Sample weights of 35–75 mg of lyophilized tissues were used. The results are presented in form of concentrations (range of µg/kg) related to the tissue dry weight and show variations between the individual patients and organs [17]. A study for determining metals in human organ tissue exposed to implant materials was presented by Swiatkowska et al. [18]. Different measurement techniques and experiments were performed using optical microscopy, laser ablation ICP-MS, synchrotron techniques such as micro X-ray fluorescence (µ-XRF) elemental mapping, and microfocus X-ray absorption spectroscopy (µ-XAS). The results of the different methods were compared to each other [18].

An experimental setup was developed and presented by Baxmann et al. to examine the susceptibility of hip junctions to fractures, corrosion as well as the release of metal particles and metal ions [15]. The concentrations of cobalt (Co), chromium (Cr) and titanium (Ti) in the test solutions were determined using ICP-OES and ICP-MS, whereby limits of detection lower than 1 µg/L were reached [15].

The performance and application forms of ICP-MS in the determination of trace elements in body fluids and tissues, as well as typically appearing interferences, are summarized by Vanhoe et al. [22,23]. The ICP-MS is a powerful measurement technique enabling low detection limits, high precision and fast measurement times. Kerger et al. demonstrated the advantages of ICP-MS measurements with interference removal using a dynamic reaction cell and alternatively a collision reaction cell especially for the determination of cobalt (Co) concentrations lower than 5 µg/L [24,25]. Furthermore, the authors stated the need for a standardized methodology for metal—especially Co—determination in human tissues using ICP-MS which is up to now not available (blood in particular) [25].

In conclusion of this literature review, an ICP-MS equipped with a collision reaction cell for interference removal was selected to determine trace metals in human tissue within the scope of this study. The examples from the literature also illustrate the complex sample preparation of tissue samples. In the scope of large studies with a high sample number, manual procedures limit the sample throughput and the accuracy (precision and trueness). Furthermore, human operators work with toxic or harmful chemical reagents. Robot-assisted measurement systems are a promising solution to overcome the limitations of manual sample preparation and to increase the operational safety and health of the laboratory staff. In this study, a measurement procedure was developed for the determination of metal traces in human tissues. The manual process including sample preparation, measurement using ICP-MS, and data evaluation was validated using certified reference material. This process was transferred to a robot-assisted automation system, which was adapted to the special requirements of this application. The automated procedure was also validated using the same reference material, and the results were compared with the manual process. In the last part of this study, metal concentrations were determined in real human tissue samples using the robotic system.

## 3. System Concept and System Design

### 3.1. General Measurement Process

**Figure 1:**
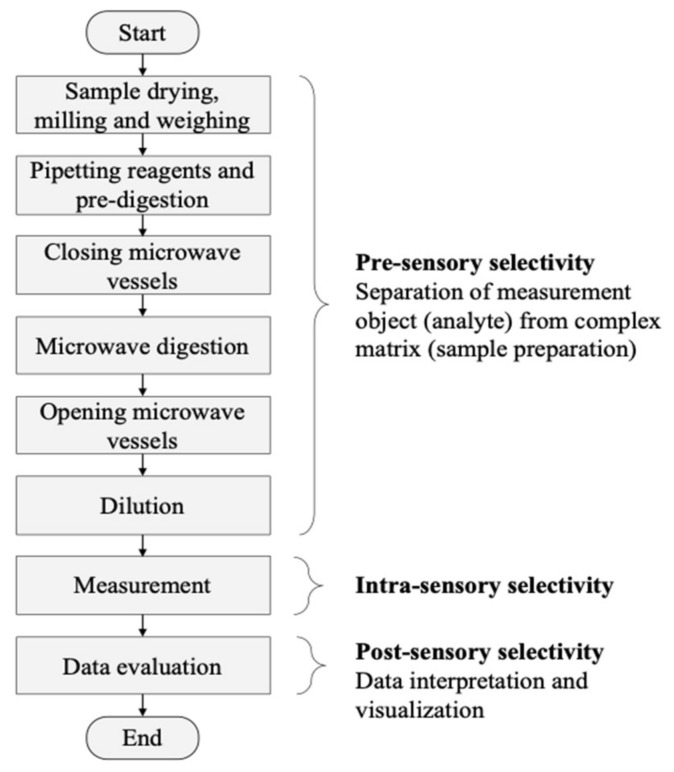
Process structure of the entire measurement process of heavy metal determination in human autopsy tissues including sample preparation, measurement and data evaluation.

Compound-oriented measurements such as the determination of heavy metals in human tissue follow the concept of pre-, intra- and post-sensory selectivity [26]. The pre-sensory selectivity will be reached by the separation of the desired elements from the complex tissue matrix. This includes sampling, drying, homogenizing by milling, weighing, transfer in solution by digestion and a final dilution. The desired analytes are separated from the complex matrix prior the sensor system. The intra-sensory selectivity can be reached by a suitable single-sensor or a complex sensor system. In this study, heavy metals were determined in human tissues using ICP-MS, whereby the metal ions are separated according to their mass–charge ratio (m/z). The final step is to achieve the post-sensory selectivity by evaluation, interpretation, and visualization of the data delivered by the mass spectrometer. Figure 1 gives an overview of the general steps in the entire measurement process for the determination of heavy metals in human tissues.

There are different ways to describe elemental concentrations in tissue such as the concentration in units of mg/kg or µg/g of ash, dry weight, or wet weight. The amount of water within tissues may vary resulting in a high variability of the element concentrations. The results of this study are reported in relationship to the sample dry weight according to [19].

### 3.2. Robot-Assisted Measurement System

Robot-assisted measurement systems can be realized in various automation concepts [27,28]. The selection of the best fitting concept to the desired application depends on the measurement process including sample preparation steps, the available laboratory space as well as on the required flexibility of the system. The automation system involved in this study has been developed as a flexible adaptable system for the application in both, elemental and structural compound-oriented measurements. It follows the concept of the central system integrator at one automation island and is involved in a decentralized structure to connect several laboratories by mobile robots [27]. Up to now, this automation system was adapted in several elemental measurement processes such as the determination of mercury in waste wood, of calcium and phosphor in bones as well as trace metals in incrustations of biliary endoprostheses and pig bile [29,30,31]. Due to the decentral interconnection to multiple measurement systems, the determination of cholesterol in incrustations of biliary stents and pig bile as well as the determination of benzoic acids were also realized [32,33]. The automation system for the robot-assisted sample preparation was reconfigured and adapted to realize the automated determination of heavy metals in human tissues presented in this study.

**Figure 2:**
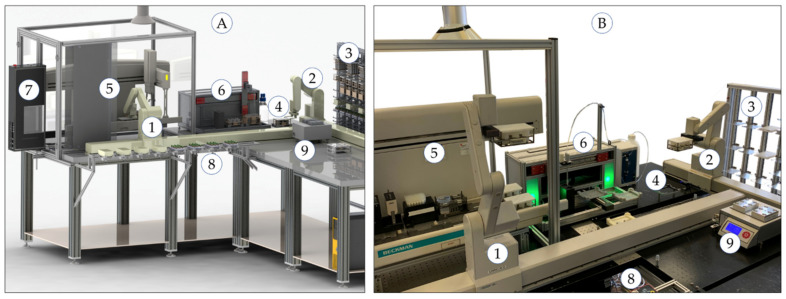
Automation system for robot-assisted sample preparation and ICP-MS measurements: 3D CAD drawing (**A**) and realized system (**B**) with (1) and (2) laboratory robots moving on two orthogonal linear rails, (3) storage system providing chemicals and labware, (4) labware transfer station between two robots, (5) liquid handler Biomek 2000, safety housing and exhaust system, (6) single-vial liquid handler, (7) positive pressure unit, (8) labware transfer station to humans or mobile robots, (9) thermo shaker.

The central elements of the automation system are two ORCA laboratory robots (Beckman Coulter, Krefeld, Germany), which are arranged on two orthogonal linear rails. This allows a maximum area of robotic action to connect individual stations by transporting labware, chemicals and samples. A storage system with 196 positions for labware in standardized microplate format can be accessed by one robot to provide all materials required in the measurement process. The pipetting of 2 mL nitric acid to the solid samples followed by 20 min rest with open vessels (pre-digestion) and the transfer of 500 µL digestion solutions into ultrapure water (dilution) is performed using the liquid handler Biomek 2000 (Beckman Coulter, Krefeld, Germany) which enables liquid volumes up to 1000 µL. A volume of 5.75 mL ultrapure water is transferred into empty vials (vol. 8 mL) using the in-house developed single vial liquid handler which enables the dosing of larger volumes up to 10 mL. The microwave digestion is performed using the device Mars 5 (CEM, Kamp-Lintfort, Germany) and the measurements are done using the ICP-MS 7700x (Agilent Technologies, Waldbronn, Germany). Both instruments are located in different laboratories. The labware and sample transport can be performed by both, laboratory assistants and mobile robots. Therefore, the labware and samples are placed on a labware transfer station in front of the automation system. The 3D CAD draft and the realized robotic system are shown in Figure 2.

**Figure 3:**
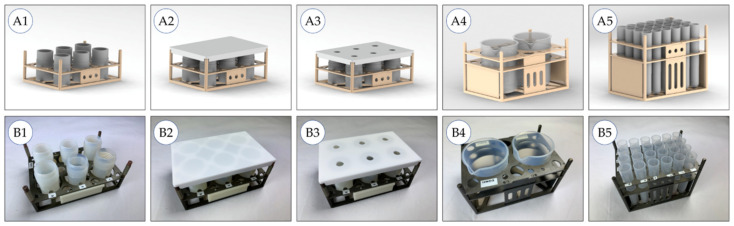
Racks in microplate format with labware: (**A**) CAD draft, (**B**) realized racks, (1) rack for six microwave vessels (vol. 10 mL), (2) lidded rack for microwave vessels, (3) lidded rack for microwave vessels with holes, (4) rack for two beakers providing the digestion reagent nitric acid (vol. 100 mL), (5) rack with vials for 24 diluted samples (vol. 15 mL).

In general, the labware used in analytical measurement processes does not have the standardized microplate format but rather has different shapes and volumes such as microwave digestion vessels, flasks, beakers and tubes. To allow the robots to handle such special labware, racks made of aluminum were designed to arrange multiple labware in the microplate footprint (Figure 3).

### 3.3. Manual and Automated Processing

The measurement process is divided into individual subprocesses including transportation steps, sample preparation and ICP-MS measurement. The entire workflow is controlled by a high-level hierarchical workflow management system (HWMS) [34]. It performs the scheduling and the control of automated processes running on robotic systems and measurement systems. Furthermore, the HWMS manages actions on the automation systems as well as interactions with human operators and mobile transportation robots [35].

In the subprocess “Initial Transport”, labware, chemicals, reagents and samples required in the measurement process are provided on the workbench and the storage positions on the automation system. The subprocess “Pipetting Acid” is automatically performed on the robotic system and involves the transport of the racks containing nitric acid in beakers and solid samples in microwave digestions vessels to the pipetting platform, the pipetting of nitric acid to solid samples and a waiting time of 20 min with open vessels (pre-digestion). The subprocess “Microwave Digestion” is supported by a human operator and involves the closing of the microwave vessels with screw caps, running the digestion procedure in the microwave digestion device and finally the opening of the microwave vessels. The subprocess “Dispensing Water” is automatically performed on the robotic system in parallel to the subprocess “Microwave Digestion” and transfers water into the vials for the final measurement solutions. The subprocess “Dilution” is also automatically performed on the robotic system and involves the pipetting of the digested sample solutions into the vials with water. The subprocess “Measurement” is performed on an ICP-MS with an automated sample introduction. The “Transportation” subprocesses involve the labware transfer between manual workbench, automated robotic system, microwave digestion device, and measurement system. The following tables summarize and highlight the differences in manual and automated processing related to the process structure (Table 1) and the required labware (Table 2).

**Figure 4:**
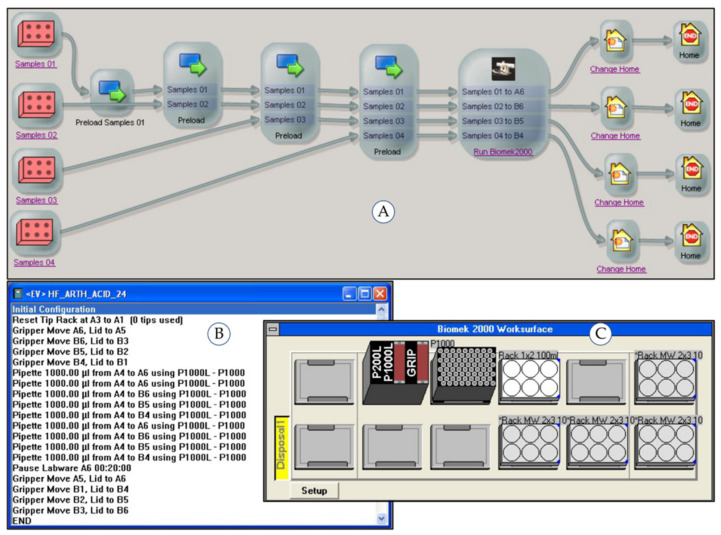
Automated subprocess “Pipetting Acid”: (**A**) SAMI Editor with process flow, (**B**) Biomek 2000 Software with pipetting method, (**C**) deck layout of liquid handler Biomek 2000.

**Figure 5:**
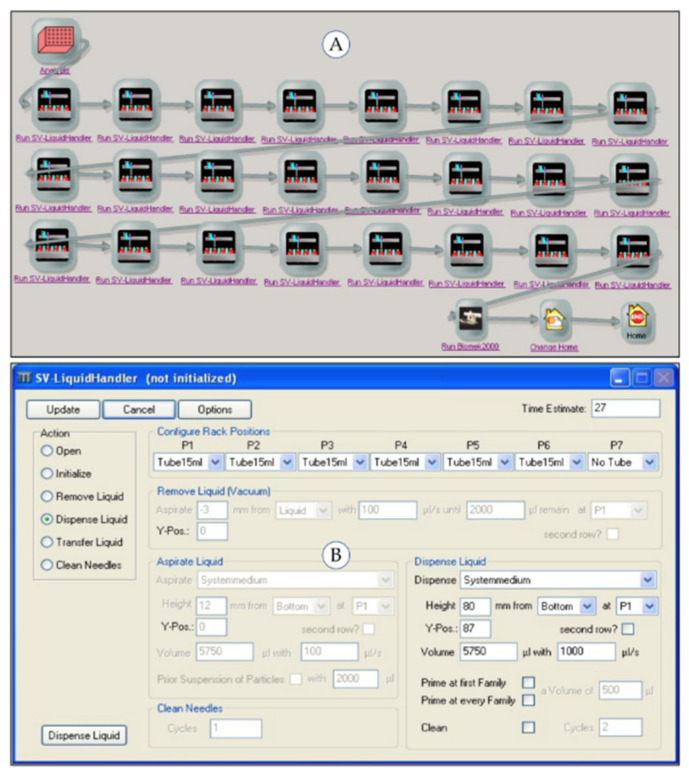
Automated subprocess “Dispensing Water”: (**A**) SAMI Editor with process flow, (**B**) inhouse software of single-vial liquid handler with dispensing parameters.

**Figure 6:**
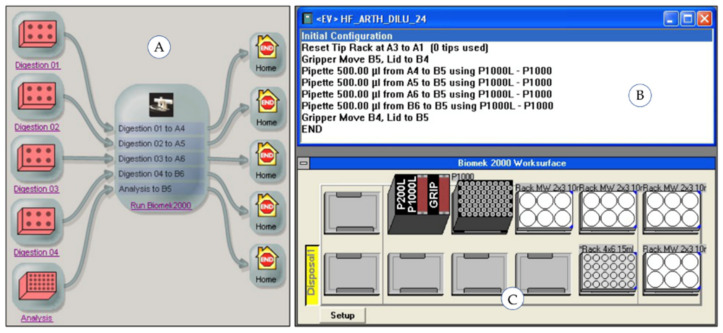
Automated subprocess “Dilution”: (**A**) SAMI Editor with process flow, (**B**) Biomek 2000 Software with pipetting method, (**C**) deck layout of liquid handler Biomek 2000.

The subprocesses running on the robotic system (“Pipetting Acid”, “Dispensing Water”, “Dilution”) were controlled using the process control software “SAMI Workstation Ex 4.0” (Beckman Coulter, Krefeld, Germany). The workflow of each automated subprocess is generated using the SAMI Editor and saved in a SAMI method. The operator configures the labware (type, source and destination positions) and the parameter of the individual substations using the respective device software. The liquid handler Biomek 2000 is controlled by the Biomek 2000 Software (Beckman Coulter, Krefeld, Germany) enabling the definition of the deck layout and the detailed pipetting parameters. The single vial-liquid handler uses an in-house built software module. A maximum of 24 samples arranged on four racks can be simultaneously processed in one SAMI method run. Multiple runs increase the sample throughput when enough starting material including samples, chemicals and labware is provided. Figure 4, Figure 5 and Figure 6 show the SAMI methods created in the SAMI Editor for the automated subprocesses and the configuration parameters of the individual substations.

## 4. Results

### 4.1. Determination of Heavy Metals in Tissue Samples

The measurement process was performed in both, the manual procedure and the automated procedure using the robotic system. The detailed materials and method descriptions are provided in Section 7. Each measurement procedure was separately validated using certified reference material NRC-BOVM-1 (Labmix24, Hamminkeln, Germany) to compare the performance (precision, trueness, and processing time) [36].

The validation experiments were similarly performed for the manual and the automated procedure and involve the determination of characteristic validation parameters [27,29,37]. The repeatability gives information about the precision within one day. Therefore, 23 CRM samples and one blank sample (nitric acid with internal standard, no analytes) were prepared and measured on the same day. These measurements were also used to determine the recovery rates calculated in relation to the elemental concentrations of the certified reference material (see Table 3). The recovery rate gives information about the trueness of the validated measurement method. The within-laboratory precision was determined with 11 CRM samples and one blank sample prepared on five consecutive days and gives information about the day-to-day precision within the same laboratory and the same equipment. The measurement precision was determined with one sample measured ten times and gives information about the precision of the ICP-MS measurement device. The limit of detection (LOD) and the limit of quantification (LOQ) gives information about the smallest detectable and the smallest quantifiable element concentration. The analytical LOD and LOQ values are related to the liquid measurement solutions and the methodological LOD and LOQ values to the solid tissue samples (dry matter). The LOD and LOQ values were determined by preparation and measurement of 12 blank samples and were calculated according to Equations (1) and (2) [38,39].

LOD = average + 3 × standard deviation(1)

LOQ = average + 10 × standard deviation(2)

The sample numbers were similarly predefined for both, the manual and the automated procedure. All vessels are handled in the automated procedure on racks. The repeatability and the recovery rate were determined with 23 CRM samples and one blank sample on four racks each with six vessels. The within-laboratory precision were determined with 11 CRM samples and one blank sample on two racks each with six vessels. The LOD and LOQ values were determined with 12 blank samples on two racks each with six vessels.

**Figure 7:**
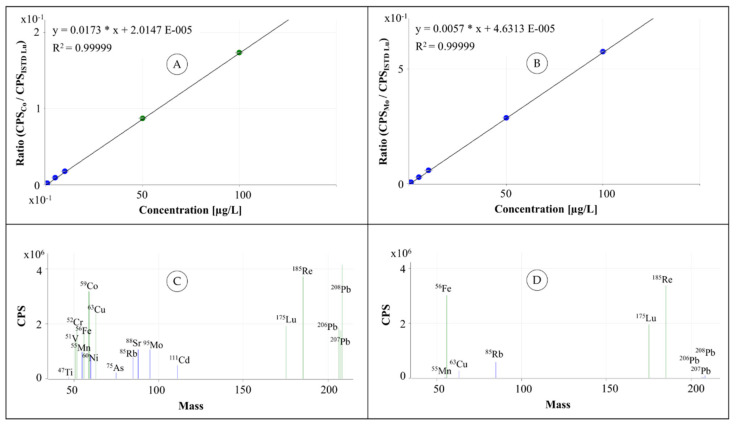
Selected ICP-MS calibrations and mass spectra acquired with collision cell helium flow of 4.3 mL/min (He tuning mode): (**A**) calibration for cobalt; (**B**) calibration for molybdenum; (**C**) mass spectrum of a calibration standard (100 µg/L); (**D**) mass spectrum of certified reference material NRC-BOVM-1.

The materials and the detailed sample preparation and ICP-MS measurement parameters are given in Section 7 (Materials and Methods). Figure 7 shows two typical calibrations of implant materials (cobalt and molybdenum), a mass spectrum of a calibration standard (concentration 100 µg/L), and a sample solution of the certified reference material NRC-BOVM-1 (both acquired with collision cell helium flow of 4.3 mL/min).

### 4.2. Validation Results of the Manual Measurement Process

The elements B, Mn, Fe, Co, Cu, Rb, Mo, Cd, and Pb were evaluated within the validation experiments. The results for the manual sample preparation and measurement process are summarized in Table 3. In general, very careful and clean working is required in the sample preparation steps to prevent contamination of the samples with elements from the surrounding environment. For example, aluminum, iron, copper, nickel and lead can be easily introduced by the surrounding laboratory environment, valves and accessories. Especially if low concentrations are expected in the samples, attention should be paid to careful and clean working.

The elements Al, Ti, V, Cr, and Ni are also from the interest in the determination of metal traces in human tissue. In the certified reference material, only information values without a measurement uncertainty (Al, V, Cr, and Ni) or no value (Ti) are given. These elements were additionally measured and selected results are presented in Table 4. It can be seen, that these element concentrations are very low. For vanadium, the methodological limit of detection (0.012 mg/kg) and limit of quantification (0.031 mg/kg) are higher than the information value (0.005 mg/kg) given for the certified reference material NRC-BOVM-1. Furthermore, no measurement uncertainty is given in the certificate. Therefore, the recovery rate of vanadium cannot be calculated within the low concentrations in the reference material. The element concentrations of aluminum, chromium and nickel are slightly higher than the methodological limit of quantification and the average concentrations of the repeatability are in accordance with the information value of the reference material, so that these elements can also be measured using the method presented.

### 4.3. Validation Results of the Automated Measurement Method

Similar elements as in the manual measurement process were determined in the validation experiments. Here, again, a very careful and clean working is required in the sample preparation steps to prevent contamination of the samples with elements from the surrounding environment. Since multiple mechanical parts are made of steel and metal alloys, aluminum (Al), chromium (Cr), iron (Fe), copper (Cu), nickel (Ni) and lead (Pb) can be easily introduced. Sources of additional metal intake—such as corroded metal parts of the racks—were found during the test measurements and were eliminated. In the case of metal parts on the racks, silicone was used to prevent metal oxide abrasion. The piston of the pipetting tool of the liquid have been serviced in shorter time frames (each week) and pipetting tips with a filter inside were used to prevent metal intake during liquid handling. The results of the automated sample preparation and measurement process performed on the robotic system are summarized in Table 5.

The elements Al, Ti, V, Cr, and Ni were additionally measured using the automated procedure. Selected results are presented in Table 6, which were for chromium and nickel in good accordance with the results from the manual procedure. Similarly, for vanadium, the methodological limit of detection (0.016 mg/kg) and limit of quantification (0.044 mg/kg) are higher than the information value (0.005 mg/kg) given for the certified reference material NRC-BOVM-1. Furthermore, no measurement uncertainty is given in the certificate. Therefore, the recovery rate of vanadium cannot be calculated within the low concentrations in the reference material. The analytical and methodological limits of detection and quantification of boron, aluminum and titanium are higher than in the manual method. Reasons can be found in a higher background caused by the mechanical parts made of metals and metal alloys of the robotic system. To determine very low concentrations of these elements, the manual procedure may be preferred.

## 5. Comparison of Manual and Automated Measurement Processes and Discussion

### 5.1. Precision and Trueness

**Figure 8:**
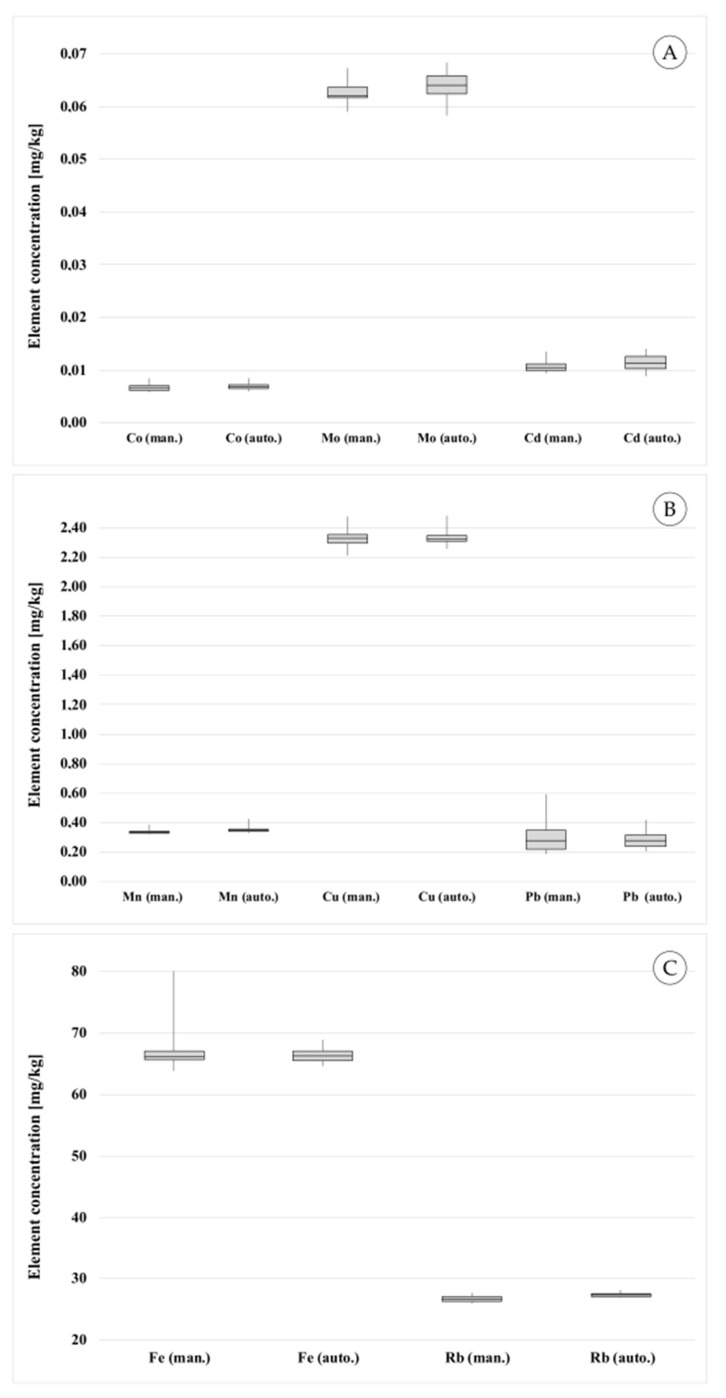
Comparison of the manual and the automated measurement process: Box-Whisker plots for (**A**) very low concentrated elements (Co, Mo, Cd), (**B**) low concentrated elements (Mn, Co, Pb), (**C**) higher concentrated elements (Fe, Rb).

The validation results presented in Section 4 show good accordance between the manual and the automated measurement processes. The precision and trueness (recovery rates) are nearly similar for the manual and the automated procedure. Figure 8 visualizes and compares the results for selected elements acquired using the manual and the automated measurement process. One Box-Whisker plot is drawn for very low concentrated (<1 mg/kg) elements (Co, Mo, Cd), one for low concentrated (<5 mg/kg) elements (Mn, Cu, Pb), and one for higher concentrated (<100 mg/kg) elements (Fe, Rb). The results confirm the successful transfer of the manual measurement process to the automation system.

### 5.2. Processing Times

A comparison of the processing times of the manual and the automated measurement process requires a detailed look inside the process. The automated procedures in 27.5 min longer. This mainly results from the pipetting steps. The automated pipetting of HNO_3_ requires 14 min more time compared to the human operator. Due to the limited maximum volume of the liquid handler Biomek 2000 (max. 1 mL) two pipetting steps are required to transfer 2 mL HNO_3_ instead of one step. The pipetting speed is lower than in manual pipetting to avoid contact between the acid and the filter inside the pipetting tip (maximum volume is aspirated). The water dispensing step requires 11.5 min longer than the human procedure due to the dispensing speed of the single-vial liquid handler. In manual processing, a microwave vessel was opened and the digestion solution aspirated and dispensed in water. In automated processing, all microwave vessels were opened and arranged in a lidded rack. Then, the liquid handler Biomek 2000 performs the pipetting procedure. This results in nearly similar processing times for this step. Table 7 gives an overview of the processing times of each process step in the manual and the automated procedure.

## 6. Determination of Heavy Metals in Human Tissue from Different Origins

### 6.1. Sample Preparation

Autopsy tissue samples were taken from nine different organs (brain, heart, lung, kidney, liver, joint capsule, lymph node, fatty tissues, and bone marrow) of five deceased. All tissues of one deceased are named as a sample set. The tissue samples were dried until constant weight and the results (element concentrations) are related to the dry matter. The detailed materials and methods parameters are provided in Section 7.

### 6.2. Results

**Figure 9:**
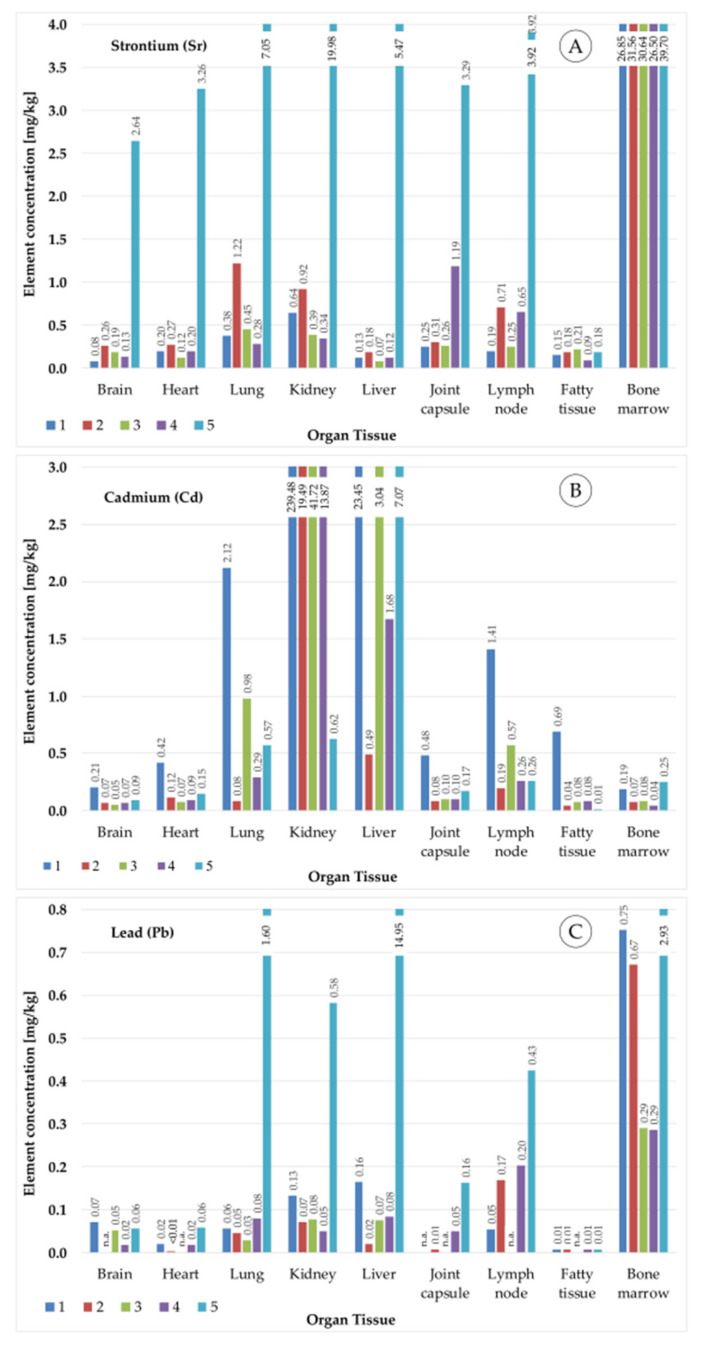
Results of measurements of tissue from different organs from five deceased (indicated with numbers from 1–5): The results (element concentrations) are related to the tissue dry matter and show the concentrations of (**A**) strontium (Sr), (**B**) cadmium (Cd), and (**C**) lead (Pb).

The results show different element concentrations depending on the organs and differences between the deceased. This study is not focused on the evaluation of the element concentrations measured in the sample sets. In this study, an automated method for the determination of metal concentrations was developed, validated and compared with the manual measurement method. The results of the measurement of real human tissue samples show the usability of the developed manual and automated measurement methods. Figure 9 shows the distribution of the three elements strontium (Sr), cadmium (Cd) and lead (Pb) within five sample sets.

In the further progress of this study, more sample sets will be measured to provide a high number of data for the investigation of the effect of metals in the human body caused by hip and knee implants. Especially the individual data of the deceased will be included in the evaluation.

## 7. Materials and Methods

### 7.1. Sample Preparation

The tissue samples were dried at 40 °C (104 °F) in a drying chamber UM 400 (Memmert, Schwabach, Germany) until constant weight. The dried tissue was homogenized by milling in ceramic containers using an oscillating ball mill MM 2000 (Retsch, Haan, Germany). The certified reference material NRC-BOVM-1 (Labmix24, Hamminkeln, Germany) was directly used after gentle shaking. Approximately 60 mg of the solid sample were transferred into a microwave vessel Xpress with a total volume of 10 mL (CEM, Kamp-Lintfort, Germany). Nitric acid in suprapure quality (Rotipuran^(R)^Supra, Carl-Roth, Karlsruhe, Germany) was used as digestion acid. To correct possible vaporization effects during the microwave-assisted acid digestion, an internal standard (ISTD2) Rhenium (Re) (ICP single-element standard, Merck, Darmstadt, Germany) was added to the digestion acid in a concentration of 1 mg/L. After sample processing, the Re concentration is about 80 µg/L in the measurement solution. A volume of 2 mL of the digestion acid with ISTD2 is added to the samples in the microwave digestion vessels. After a rest of 20 min (pre-digestion) with open vessels under the exhaust system, three PTFE agitator balls (diameter 6 mm, Bola, Grünsfeld, Germany) were added to the vessels and the vessels were closed. The microwave digestion was performed using the following parameters: temperature-time ramp to 150 °C (356 °F) in 20 min with a final hold time of 20 min at 150 °C (356 °F) with 100% power of 600W. After cooling down to room temperature, the vessels were opened and the samples dissolved with ultrapure water (1:12.5, *v*/*v*).

### 7.2. Measurement of Aqueous Sample Solutions Using ICP-MS

The calibration was created using the ICP multi-element standard IV and ICP single-element standards of Re, V, Be, As, Mo, Ti and Rb each with an element concentration of 1000 mg/L (Merck, Darmstadt, Germany). The calibration for determining very low concentrations was created using nine calibration standards with element concentrations of 0.01, 0.05, 0.1, 0.5, 1, 5, 10, 50 and 100 µg/L. A typical calibration for the normal ICP-MS working range is created only with five standards of 1, 5, 10, 50 and 100 µg/L. To correct variations in the sample introduction, the internal standard Lu (ISTD 1) with a concentration of 500 µg/L was aspirated by the peristaltic pump, mixed with the sample solution using a T-piece and introduced. The measurements of the diluted sample solutions were performed using an ICP-MS 7700x with the MassHunter 4.4 Workstation Software for 7700 ICP-MS G7201C Version C.01.04 Build 544.3 (Agilent Technologies, Waldbronn, Germany). The following measurement conditions were applied: argon as plasma and nebulizer gas, RF power 1550 W, sample depth 10 mm, nebulizer gas flow 1.05 L/min, nebulizer pump 0.10 rps, spray chamber temperature 13 °C (55.4 °F), collision cell with no helium flow in NoGas tuning mode and 4.3 mL/min helium flow in He tuning mode. Each measurement was performed with three repetitions and the following acquisition parameters: ^11^B, ^27^Al and ^175^Lu (ISTD 1) in NoGas mode with an integration time of 0.1 s (^11^B, ^27^Al) and 0.3 s (^175^Lu) as well as ^47^Ti, ^51^V, ^52^Cr, ^55^Mn, ^58^Fe, ^60^Ni, ^63^Cu, ^66^Zn, ^75^As, ^85^Rb, ^88^Sr, ^95^Mo, ^107^Ag, ^111^Cd, ^175^Lu (ISTD 1), ^185^Re (ISTD 2), and ^206,207,208^Pb in He mode with an integration time of 0.3 s. The sample flush time was 10 sec with a pump speed of 0.1 rps. The flushing times between two samples to prevent cross contamination were 55 sec at 0.3 rps with HNO_3_/HCl (5%/1%, *v*/*v*) and 45 sec at 0.4 rps with HNO_3_ (8%, *v*/*v*). The automated sample introduction was performed using the autosampler ASX-500 (Cetac, Omaha, NE, USA).

## 8. Conclusions

A robot-assisted measurement system is presented for the semi-automated determination of heavy metals in human tissue samples. The manual and the automated measurement processes were similarly validated using certified reference material and the results were compared. The system allows the pre-processing and measurement of a high number of samples with similar precision and trueness compared to manual processing. The total processing time of the automated procedure requires approx. 11% more time than the manual procedure. The system can be further optimized by simultaneous execution of different tasks for several sample batches. During the microwave digestion, the pre-digestion for the next sample batch could take place. This would not reduce the processing time for a sample set, but would allow continuous use of the system. Overall, this would enable a higher sample throughput. In this case, a workflow management system is required that takes over the scheduling of the individual tasks. Another positive effect of automation is, that human operators can take over other tasks in the lab increasing the overall laboratory performance and productivity. Furthermore, the laboratory staff is prevented to work with toxic and harmful chemicals and a high manual workload increasing safety and health.

The manual process was successfully transferred to the automation system and enables the processing of a high sample throughput. Therefore, the semi-automated robot-assisted determination of metals in human tissues may be a powerful driver of the current research into the exploration of negative side effects of implant materials to human health.

## Figures and Tables

**Table 1:** Comparison of process steps performed in the manual and in the automated sample preparation and measurement process.

Subprocess	Process Step	Manual	Automated
Initial Transport	Transport by human	Samples, labware, reagents to workbench	Samples, labware, reagents to storage system
Pipetting Acid	Transport to liquid handler Biomek2000	-	Robots ORCA 1 and ORCA 2
	Pipetting HNO_3_	2 mL/sample	2 × 1 mL/sample
	Pre-digestion	Rest of 20 min	Rest of 20 min
	Put lid on rack	-	Biomek2000
	Transport to transfer station	-	Robot ORCA 2
Transport	Transport to workbench	-	Human or mobile robot
Microwave Digestion	Remove vessels from rack	-	Human
	Close microwave vessels	Human	Human
	Transport into microwave, run digestion	Human	Human
	Open microwave vessels	Human	Human
	Put microwave vessels into rack (punched lid)	-	Human
Transport	Transport to automation system	-	Human or mobile robot
Dispensing Water	Transport to single-vial liquid handler	-	Robots ORCA 1 and ORCA 2
	Remove lid from rack	-	Robot ORCA 2
	Pipetting water	11.5 mL/sample	5.75 mL/sample
	Put lid on rack	-	Robot ORCA 2
	Transport to Biomek 2000	-	Robot ORCA 2
Dilution	Remove lid from rack	-	Biomek2000
	Pipetting digestion solution	1 mL/sample	0.5 mL/sample
	Put lid on rack	-	Biomek2000
	Transport to transfer station	-	Robot ORCA 2
Transport	Transport to ICP-MS	Human	Human or mobile robot
Measurement	ICP-MS measurement	ICP-MS	ICP-MS
Final Transport	Waste disposal, cleaning	Human	Human or mobile robot

**Table 2:** Comparison of labware used in the manual and in the automated procedure for the preparation and measurement of 24 tissue samples.

Labware	Used in	
	Manual Process	Automated Process
Microwave vessel Xpress (vol. 10 mL)	24 vessels	24 vessels
Rack for six microwave vessels	-	4 racks
Solid lid for rack to cover 6 microwave vessels	-	4 solid lids
Punched lid with 6 holes for safe pipetting	-	4 punched lids
Beaker for nitric acid (vol. 100 mL)	1 beaker	1 beaker
Rack for 2 beakers (1 used)	-	1 rack
Tubes for diluted sample solutions (vol. 15 mL)	24 tubes (screw caps)	24 open tubes
Plastic rack for tubes (screw caps, vol. 15 mL)	1 rack	-
Rack for 24 tubes (vol. 15 mL)	-	1 rack
Solid lid for rack to cover 24 tubes	-	1 solid lid

**Table 3:** Validation results for selected elements acquired using the manual measurement process and reference values of NRC-BOVM-1 [36].

	B	Mn	Fe	Co	Cu	Rb	Mo	Cd	Pb
Repeatability (n = 23)									
Average (mg/kg)	0.240	0.339	67.207	0.007	2.329	26.765	0.063	0.011	0.311
STDEV (mg/kg)	0.029	0.016	3.927	0.001	0.058	0.475	0.002	0.001	0.125
CV (%)	12.0	4.7	5.8	10.6	2.5	1.8	3.0	9.6	40.2
Recovery rate (n = 23) (related to the average reference value of NRC-BOVM-1)									
Average (%)	40.0	91.7	94.4	96.2	82.0	93.3	78.4	81.9	81.8
STDEV (%)	4.8	4.3	5.5	10.2	2.1	1.7	2.3	7.8	32.9
CV (%)	12.0	4.7	5.8	10.6	2.5	1.8	3.0	9.6	40.2
Min (%)	36.0	86.4	89.6	83.6	77.8	90.7	73.9	72.0	49.0
Max (%)	60.3	104.3	118.1	120.6	87.2	96.8	84.1	105.7	155.6
Within-laboratory precision (at five days with n = 11)									
Min. Av. (mg/kg)	0.218	0.316	63.666	0.007	2.263	26.014	0.062	0.011	0.278
Max. Av. (mg/kg)	0.277	0.367	69.012	0.008	2.671	28.466	0.066	0.013	0.328
Min CV (%)	4.3	2.3	1.3	5.8	2.5	1.2	2.9	10.0	17.2
Max CV (%)	11.9	7.0	4.2	17.2	21.5	4.1	4.6	32.6	49.7
Measurement precision (n = 1 with 10 measurements)									
CV (%)	4.1	1.3	1.2	5.9	1.0	0.8	3.6	14.6	0.7
Analytical LOD and LOQ (in measurement solution)									
LOD (µg/L)	0.163	0.049	0.904	0.003	0.229	0.026	0.006	0.004	0.010
LOQ (µg/L)	0.418	0.127	2.485	0.005	0.552	0.063	0.016	0.010	0.021
Methodological LOD and LOQ (in dried tissue samples)									
LOD (mg/kg)	0.068	0.020	0.377	0.001	0.096	0.011	0.003	0.002	0.004
LOQ (mg/kg)	0.174	0.053	1.035	0.002	0.230	0.026	0.007	0.004	0.009
Reference values of NRC-BOVM-1									
Average (mg/kg)	0.6	0.37	71.2	0.007	2.84	28.7	0.08	0.013	0.38
Uncertainty (mg/kg)	±0.4	±0.09	±9.2	±0.003	±0.45	±3.5	±0.06	±0.011	±0.24
Uncertainty (%)	66.7	24.3	12.9	42.9	15.8	12.2	75.0	84.6	63.2

**Table 4:** Results for additional elements acquired using the manual measurement process and respective information values of NRC-BOVM-1 [36].

	Al	Ti	V	Cr	Ni
Repeatability (n = 23)					
Average (mg/kg)	1.660	0.831	0.035	0.064	0.032
STDEV (mg/kg)	0.556	0.601	0.007	0.026	0.008
CV (%)	33.5	72.4	19.6	40.1	23.9
Recovery rate (n = 23) (related to the average information value of NRC-BOVM-1)					
Average (%)	97.7	-	<LOD	90.5	64.2
STDEV (%)	32.7	-	<LOD	36.4	15.4
CV (%)	33.5	-	<LOD	40.1	23.9
Min (%)	62.3	-	<LOD	52.6	43.3
Max (%)	183.6	-	<LOD	198.2	110.5
Analytical LOD and LOQ (in measurement solution)					
LOD (µg/L)	1.655	0.421	0.030	0.036	0.030
LOQ (µg/L)	3.732	1.135	0.074	0.101	0.081
Methodological LOD and LOQ (in dried tissue samples)					
LOD (mg/kg)	0.690	0.175	0.012	0.015	0.013
LOQ (mg/kg)	1.555	0.473	0.031	0.042	0.034
Information values of NRC-BOVM-1					
Average (mg/kg)	1.700	n.a.	0.005	0.071	0.050

**Table 5:** Validation results for selected elements acquired using the automated measurement process and reference values of NRC-BOVM-1 [36].

	B	Mn	Fe	Co	Cu	Rb	Mo	Cd	Pb
Repeatability (n = 23)									
Average (mg/kg)	0.173	0.349	66.382	0.007	2.330	27.418	0.064	0.012	0.290
STDEV (mg/kg)	0.022	0.018	1.171	0.001	0.043	0.294	0.003	0.001	0.064
CV (%)	12.9	5.1	1.8	8.7	1.8	1.1	4.2	12.7	21.9
Recovery rate (n = 23) (related to the average reference value of NRC-BOVM-1)									
Average (%)	28.8	94.3	93.2	98.6	82.0	95.5	80.2	88.7	76.3
STDEV (%)	3.7	4.8	1.6	8.6	1.5	1.0	3.4	11.3	16.8
CV (%)	12.9	5.1	1.8	8.7	1.8	1.1	4.2	12.7	21.9
Min (%)	24.5	89.7	90.8	85.1	79.5	93.9	72.8	67.5	54.4
Max (%)	37.9	114.2	96.7	121.7	87.4	98.2	85.6	108.7	109.8
Within-laboratory precision (at five days with n = 11)									
Min. Av. (mg/kg)	0.153	0.347	65.648	0.007	2.319	27.152	0.063	0.011	0.290
Max. Av. (mg/kg)	0.192	0.360	67.286	0.008	2.578	27.442	0.069	0.013	0.426
Min CV (%)	8.6	2.6	1.2	6.7	1.0	0.8	3.5	9.9	18.1
Max CV (%)	17.4	7.1	2.3	44.4	2.8	2.4	7.3	41.1	63.1
Measurement precision (n = 1 with 10 measurements)									
CV (%)	5.5	1.8	1.1	4.8	1.3	1.6	4.2	11.1	1.0
Analytical LOD and LOQ (in measurement solution)									
LOD (µg/L)	0.262	0.053	1.681	0.004	0.206	0.011	0.028	0.006	0.015
LOQ (µg/L)	0.478	0.134	4.546	0.008	0.563	0.030	0.075	0.015	0.041
Methodological LOD and LOQ (in dried tissue samples)									
LOD (mg/kg)	0.109	0.022	0.700	0.002	0.086	0.005	0.012	0.003	0.006
LOQ (mg/kg)	0.199	0.056	1.894	0.003	0.235	0.012	0.031	0.006	0.017
Reference values of NRC-BOVM-1									
Average (mg/kg)	0.6	0.37	71.2	0.007	2.84	28.7	0.08	0.013	0.38
Uncertainty (mg/kg)	±0.4	±0.09	±9.2	±0.003	±0.45	±3.5	±0.06	±0.011	±0.24
Uncertainty (%)	66.7	24.3	12.9	42.9	15.8	12.2	75.0	84.6	63.2

**Table 6:** Validation results for selected elements acquired using the automated measurement process and information values of NRC-BOVM-1 [36].

	Al	Ti	V	Cr	Ni
Repeatability (n = 23)					
Average (mg/kg)	1.579	0.595	0.035	0.060	0.037
STDEV (mg/kg)	0.351	0.290	0.006	0.010	0.012
CV (%)	22.2	48.8	18.5	17.0	34.0
Recovery rate (n = 23) (related to the average information value of NRC-BOVM-1)					
Average (%)	92.9	-	<LOD	85.2	73.1
STDEV (%)	20.6	-	<LOD	14.5	24.9
CV (%)	22.2	-	<LOD	17.0	34.0
Min (%)	66.4	-	<LOD	66.6	41.2
Max (%)	133.5	-	<LOD	118.7	137.7
Analytical LOD and LOQ (in measurement solution)					
LOD (µg/L)	3.321	0.785	0.016	0.036	0.047
LOQ (µg/L)	7.865	2.123	0.044	0.079	0.099
Methodological LOD and LOQ (in dried tissue samples)					
LOD (mg/kg)	1.384	0.327	0.007	0.015	0.020
LOQ (mg/kg)	3.277	0.884	0.018	0.033	0.041
Information values of NRC-BOVM-1					
Average (mg/kg)	1.700	n.a.	0.005	0.071	0.050

**Table 7:** Comparison of process times for processing of 24 samples in the manual and the automated measurement procedure (minutes were rounded to full 0.5 min).

Process Step	Processing Time [min]	
	Manual	Automated
Initial transport	5.0	6.5
Pipetting HNO_3_	2.5	16.5
Pre-digestion	20.0	20.0
Close microwave vessels	5.0	5.0
Microwave digestion	70.0	70.0
Dispensing water	4.5	16.0
Open microwave vessels	26.5 ^1^	10.0
Pipetting digestion solution		17.0
ICP-MS measurements	114.0	114.0
**Total Processing Time**	**247.5**	**275.0**

^1^ In manual processing, a microwave vessel was opened and the digestion solution aspirated and dispensed in water. In automated processing, all microwave vessels were opened and arranged in a lidded rack. Then, the liquid handler performs the pipetting procedure.

## Data Availability

The data presented in this study are available on request from the corresponding author. The data are not publicly available due to privacy and ethics.

## Sample Availability

Samples of the compounds are not available from the authors.
